# Femoral versus Multiple Nerve Blocks for Analgesia after Total Knee Arthroplasty

**DOI:** 10.5041/RMMJ.10281

**Published:** 2017-01-30

**Authors:** Anatoli Stav, Leonid Reytman, Roger Sevi, Michael Yohay Stav, Devorah Powell, Yanai Dor, Mickey Dudkiewicz, Fuaz Bayadse, Ahud Sternberg, Michael Soudry

**Affiliations:** 1 Postanesthesia Care Unit, Hillel Yaffe Medical Center, Hadera, Israel; 2 The Ruth and Bruce Rappaport Faculty of Medicine, Technion-Israel Institute of Technology, Haifa, Israel; 3 Department of Anesthesiology, Hillel Yaffe Medical Center, Hadera, Israel; 4 Department of Orthopedics A, Hillel Yaffe Medical Center, Hadera, Israel; 5 Director-General, Hillel Yaffe Medical Center, Hadera, Israel; 6 Department of Surgery A, Hillel Yaffe Medical Center, Hadera, Israel

**Keywords:** Arthroplasty, opioid consumption, pain, range of motion, ultrasound-guided peripheral nerve block

## Abstract

**Background:**

The PROSPECT (Procedure-Specific Postoperative Pain Management) Group recommended a single injection femoral nerve block in 2008 as a guideline for analgesia after total knee arthroplasty. Other authors have recommended the addition of sciatic and obturator nerve blocks. The lateral femoral cutaneous nerve is also involved in pain syndrome following total knee arthroplasty. We hypothesized that preoperative blocking of all four nerves would offer superior analgesia to femoral nerve block alone.

**Methods:**

This is a prospective, randomized, controlled, and observer-blinded clinical study. A total of 107 patients were randomly assigned to one of three groups: a femoral nerve block group, a multiple nerve block group, and a control group. All patients were treated postoperatively using patient-controlled intravenous analgesia with morphine. Pain intensity at rest, during flexion and extension, and morphine consumption were compared between groups over three days.

**Results:**

A total of 90 patients completed the study protocol. Patients who received multiple nerve blocks experienced superior analgesia and had reduced morphine consumption during the postoperative period compared to the other two groups. Pain intensity during flexion was significantly lower in the “blocks” groups versus the control group. Morphine consumption was significantly higher in the control group.

**Conclusions:**

Pain relief after total knee arthroplasty immediately after surgery and on the first postoperative day was significantly superior in patients who received multiple blocks preoperatively, with morphine consumption significantly lower during this period. A preoperative femoral nerve block alone produced partial and insufficient analgesia immediately after surgery and on the first postoperative day. (Clinical trial registration number (NIH): NCT01303120)

## INTRODUCTION

The PROSPECT Working Group (postoppain.org/ working-group) published a procedure-specific review and consensus recommendations (i.e. guide-lines) for analgesia after total knee arthroplasty (TKA) in 2008 in which a single injection femoral nerve block alone was recommended.^1^ We used a preoperative single injection femoral nerve block alone as an adjuvant to patient-controlled analgesia with intravenous morphine (PCA IV MO) for analgesia after TKA from September 2010 to May 2011. We found that femoral nerve block alone produced a partial analgesic effect. Pain after TKA peaks during the 48 hours after surgery.^2^ Thirty-two different techniques for anesthesia and postoperative pain management for TKA were identified in the hospitals of South West England^3^ after publication of the PROSPECT guidelines. Many prospective randomized controlled trials, a number of reviews, and a meta-analysis have been published to determine the optimal perioperative analgesia method following TKA.^4^–^20^

The knee joint innervation includes the femoral, obturator, and sciatic nerves.^21^ The lateral femoral cutaneous nerve is involved in innervation to the lateral knee skin and in pain syndrome^17^,^21^,^22^ following TKA, and this nerve should be blocked along with the other three nerves. This study compared the efficacy of ultrasound (US)-guided preoperative femoral nerve block (FNB) alone^1^–^6^ to that of US-guided multiple preoperative nerve block (MNB) of all four nerves (femoral, sciatic, obturator, and lateral femoral cutaneous), since, to the best of our knowledge, such a comparison has not yet been performed or published.

## METHODS

This is a prospective, randomized, controlled, and observer-blinded clinical study; enrollment began after receiving Hillel Yaffe Review Board approval and written informed patient consent. Study eligibility was as follows: over the age of 18; physical status of I–III based on the American Society of Anesthesiologists criteria; and scheduled to undergo elective TKA due to osteoarthritis. In total, 120 patients were eligible for the trial.

Exclusion criteria were previous TKA, TKA revision, TKA due to trauma or etiology other than osteoarthritis, under 18 years of age, presence of a local skin infection near the block injection site, allergy to local anesthetics, pre-existing peripheral neuropathy of the involved limb, demonstrated opioid dependency,^23^ coagulopathy, chronic pain syndrome, dementia, and/or an inability to comprehend the pain scale or use the PCA IV MO device.

Patients were instructed regarding the use of a 100 mm visual analogue scale (VAS) graded from 0 (without pain) to 100 (intolerable pain).

**Figure 1:**
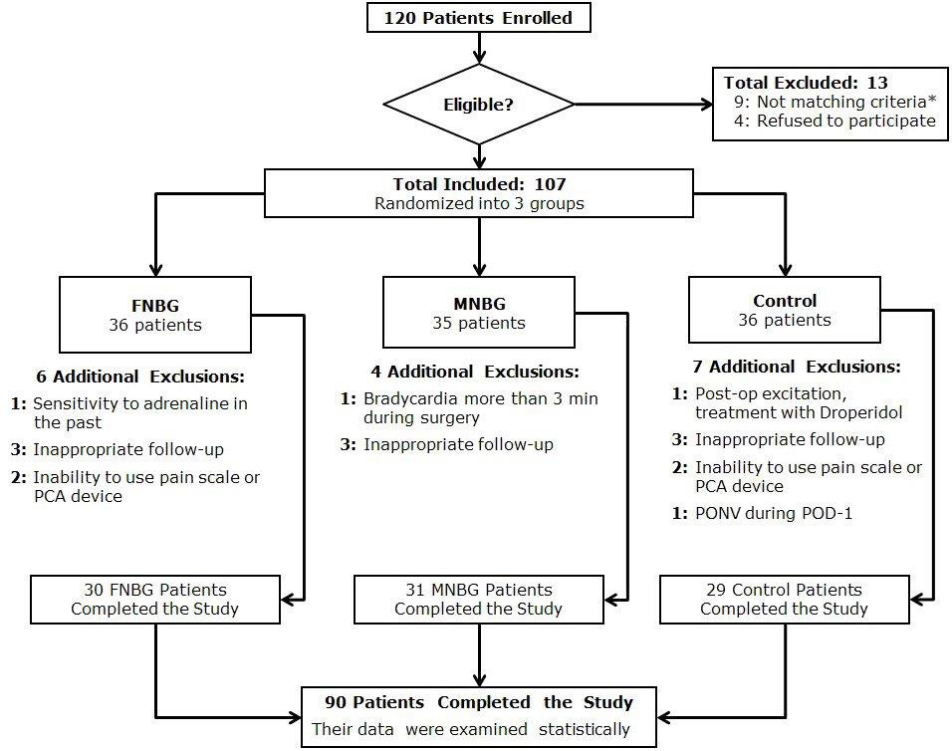
Consolidated Standards of Reporting Clinical Trials (CONSORT) Flow Chart of Included and Excluded Patients. The inclusion criteria were: physical status of I–III based on the American Society of Anesthesiologists criteria; and scheduled to undergo elective TKA due to osteoarthritis. * The exclusion criteria were: previous TKA, revision of TKA, TKA due to trauma or etiology other than osteoarthritis, age<18, a skin infection, allergy to local anesthetic, pre-existing peripheral neuropathy, opioid dependence, coagulopathy, chronic pain syndrome, dementia, inability to comprehend the pain scale or to use the PCA device. FNBG, femoral nerve block group; MNBG, multiple nerve blocks group; PCA, patient-controlled analgesia; PONV, postoperative nausea and vomiting; POD-1, first postoperative day; TKA, total knee arthroplasty.

Based on the exclusion criteria, 13 patients were excluded from the study. The inclusion group (107 patients) was randomized into three groups: femoral nerve block group (FNBG), multiple nerve block group (MNBG), and a control group (Figure 1). Randomization was done using a computer-generated table of random numbers, placing them in a sealed envelope, and then opening the envelope on the morning of surgery. Patients in the control group did not undergo a preoperative peripheral nerve block.

The patients and the investigators who collected data during the postoperative period were blinded to group assignments throughout the research.

Premedication was limited to IV fentanyl (0.5 μg/kg), midazolam (0.03 mg/kg), and local anesthesia via injection of lidocaine 10 mg/mL (3–5 mL, FNB; maximum of 15 mL, MNBG).

The same anesthesiologists (A. Stav or L.R.) performed all preoperative US-guided peripheral nerve blocks using an ultrasound system (SonoSite S-Nerve, SonoSite, Bothell, WA, USA) with a 6–13 MHz linear array transducer (L25x). A 22-gauge 80-mm Pajunk Sono Tap cannula (Pajunk® Medical Produkte GmbH, Geisingen, Germany) was used to perform the femoral, obturator, and popliteal sciatic nerve blocks. A 50-mm 22-gauge needle was used for the lateral femoral cutaneous nerve block.

Only single injection blocks were performed, to enable the initiation of physiotherapy on the first postoperative day (POD-1), since continuous peripheral nerve blocks may prevent active movement of the lower extremities.

Standard non-invasive monitors were used, and oxygen was administered via a facemask, 5 L per minute. The local anesthetic injected for nerve blocks comprised bupivacaine (5 mg/mL) with adrenaline (5 μg/mL). The femoral and sciatic nerves were blocked using 15 mL of local anesthetic, and the obturator nerve was blocked using 5 mL of the local anesthetic for each branch (the anterior and posterior branches). Lateral femoral cutaneous nerve block was performed using 5 mL of lidocaine 10 mg/mL. This block is important during the operation, but less in the postoperative period, because lateral femoral cutaneous nerve does not innervate the knee joint.

All US-guided peripheral nerve blocks were conducted using previously published techniques.^24^–^30^

The success of the sensory and motor blockade of each nerve was tested 30 min after completion of the preoperative procedure (both FNBG and MNBG) by an independent investigator who was blinded to the previously performed block. The sensory function of each of the blocked nerves was examined using a pinprick to the appropriate area. The motor function of each blocked nerve was examined by flexion ability, extension of the knee and hip joints (femoral and sciatic nerves), and adduction ability of the previously abducted lower limb (obturator nerve). Patients were additionally excluded from the study if a failed block was diagnosed (normal sensation and/or normal motor function).

Ondansetron (8 mg, IV) was injected before general anesthesia induction as prophylaxis for post-operative nausea and vomiting.^31^ A standardized general anesthesia protocol was used for all patients (total intravenous anesthesia with propofol and remifentanil^32^,^33^). All patients received 0.1 mg/kg morphine (IV) 30 min prior to the end of surgery to eliminate remifentanil-induced hyperalgesia.^34^,^35^ All operations were performed by or under the super-vision of the same experienced orthopedic surgeon (R.S.). The PCA IV MO was initiated in the post-anesthesia care unit (PACU) for all patients. The patient-controlled analgesia (PCA) protocol was as follows: a loading bolus of 2 mg IV morphine, 1 mg for the subsequent bolus, followed by a lockout period of 5 min, with a maximal dosage of 50 mg within a 4-hour period; PCA continued for three postoperative days.

Variables assessed before and during surgery included patient characteristics (age, gender, height, and body weight (BW)). Body mass index (BMI) was calculated using the following formula: BMI=BW (kg)/height^2^ (m^2^). Preoperative pain at rest and during motion was measured using a visual analogue scale from 0 to 100 mm,^36^ the preoperative range of motion in the involved joint was examined using a goniometer (degrees), and distance of walking without pain (m) and duration of surgery (min) were recorded. All were evaluated statistically. If oxygen saturation decreased during surgery, or bradycardia, low blood pressure, or other events persisted for more than 2–3 min, the patient was excluded from the study. The aim of this exclusion was to remove potential temporary intraoperative brain tissue oxygen desaturation, which may influence mental state^37^ and pain intensity estimation during the postoperative period when assessed by VAS. Pain intensity in the PACU was measured using the modified nurses’ assessment of postoperative pain scale (NP): 0, no pain or the patient is asleep; 1, mild pain or discomfort; 2, moderate pain; 3, severe pain; 4, intolerable pain.^38^

If moderate, severe, or intolerable pain was experienced by the patient in the PACU immediately postoperatively, then morphine was initially injected using a titration method until pain relief was achieved,^39^ followed by PCA IV MO. Morphine consumption in the PACU, postoperative nausea and vomiting (PONV) (yes/no), and time of stay were assessed and statistically compared between the groups. Patients were discharged from the PACU based on the revised Aldrete Scoring System.^40^ The minimum PACU duration was 30 min. In addition, data were collected after discharge from the PACU, i.e. during postoperative day-0 (POD-0), and the first (POD-1) and second postoperative days (POD-2) regarding pain intensity at rest and during flexion/extension of the involved joint (VAS), as well as range of motion flexion and extension in degrees (goniometer), walking distance with a walker (m) during POD-1 and/or POD-2 and evaluated statistically. Satisfaction with analgesia during the day of surgery and two postoperative days was measured by VAS scale principle at the end of POD-2.

The investigator collecting the data during POD-0, POD-1, and POD-2 was blinded regarding the type of previously performed block.

The primary end-points of the trial included pain at rest and morphine consumption immediately after surgery (in the PACU) and on POD-0, POD-1, and POD-2. An *a priori* power analysis was performed for these variables.

The parameters for the secondary end-point were: postoperative nausea and vomiting in the PACU (yes/no); PACU time of stay; active range of motion on POD-1 and POD-2; pain during flexion and extension on POD-1 and POD-2; walking distance using a walker on POD-1 and/or POD-2; patient satisfaction with analgesia during the evaluated postoperative period.

### Statistical Analysis

An *a priori* power analysis of primary end-points was performed using G* Power 3.0.10 (Heinrich Heine Universität Düsseldorf, Düsseldorf, Germany) with a fixed effects, omnibus, one-way ANOVA for all groups. A total sample size of 66 was considered adequate to achieve an effect size of 0.5 with an α error probability of 0.05 and a power (1-β error probability) of 0.95. After all exclusions, our study included 90 patients.

Statistical analysis was performed using IBM^®^ SPSS^®^ Statistics 20 (IBM Corporation, Armonk, NY). Continuous numerical parameters were analyzed using the Shapiro–Wilk test for normality distribution, followed by the Levene test for homogeneity of the variances (if a normal distribution was determined). Parameters with a normal distribution and homogeneous variances were compared by one-way ANOVA followed by Tukey’s *post-hoc* test, if necessary.

The Kruskal–Wallis test was used when an abnormal distribution of the continuous variables was detected. The Mann–Whitney *post-hoc* test was used following the Kruskal–Wallis test, if necessary.

Frequency tables and Pearson chi-square tests were used to compare the proportions between the categorical variables among the groups.

A value of *P*<0.05 was considered statistically significant.

## RESULTS

Study data were collected between June 13, 2011 and July 7, 2014. The results of our trial are presented according to the standards of reporting Clinical Trials (CONSORT) statement using a CONSORT flow chart (Figure 1). The data obtained from 90 patients were statistically analyzed until the end of the trial.

Preoperative variables were comparable between groups (Table 1), except BW and BMI differences between the MNBG and control group (Mann–Whitney test for comparison of continuous variables between two independent groups: *P*=0.003 and 0.005 respectively). There were no difference in surgery duration. There were no complications during or immediately after the blocks in the FNBG and MNBG.

**Table 1:** Preoperative Data.

	FNBG (*n*=30)	MNBG (*n*=31)	Control (*n*=29)	*P* Value
Age (years) *	69±1.24	69±1.56	67±1.22	0.58
Gender (M:F) †	9:21	12:19	11:18	0.74
Height (m) ‡	1.61±0.02	1.63±0.01	1.63±0.02	0.78
BW (kg) ‡	85.98±2.88	81.55±2.70	92.97±2.80	0.01
BMI (kg/m^2^) ‡	33.06±0.94	30.6±1.06	35.15±1.12	0.01
Pain VAS rest (mm) ‡,**	10.85±2.91	10.59±3.01	17.63±4.46	0.85
Flexion (centigrade) *,§	104.37±4.54	109.94±5.31	110.24±4.67	0.63
Pain VAS flexion (mm) ‡,§,**	41.54±4.95	35.71±5.24	46.82±6.76	0.45
Extension (centigrade) ‡,§	179.33±0.40	176.55±1.95	178.45±0.79	0.72
Pain VAS extension (mm) ‡,§,**	21.15±4.22	25.61±4.61	32.98±6.79	0.58
Walking without pain (m) ‡	188.17±35.97	350.45±69.81	246.38±73.18	0.25

All values are presented as mean ±SE.

* Age and flexion were of normal distribution (Shapiro–Wilk test) and homogeneous of variances (Levene test). These variables were analyzed using ANOVA test for comparison of means between three groups.

† Pearson chi-square test for proportions between categorical variables.

‡ All variables were of abnormal distribution. Therefore, the Kruskal–Wallis test was used for comparison of continuous variables between three independent groups. The Mann–Whitney *post-hoc* test was used following the Kruskal–Wallis test, if necessary.

§ Flexion or extension: maximal possible range of motion during flexion and extension were measured by goniometer in centigrade.

** Pain VAS rest (mm), Pain VAS flexion, Pain VAS extension: pain intensity at rest, during flexion, or during extension were measured by VAS in mm.

BMI (kg/m^2^), body mass index; BW (kg), body weight in kilograms; FNBG, femoral nerve block group; MNBG, multiple nerve block group; VAS, visual analogue scale.

Pain intensity in the PACU and morphine consumption were significantly lower in the MNBG than in the other two groups (Table 2). Pain relief was greater and morphine consumption was lower in the FNBG than in the control group (Table 2). These results were the primary end-points of our study.

**Table 2:** Pain Intensity and Drug Consumption.

	FNBG (*n*=30)	MNBG (*n*=31)	Control (*n*=29)	*P* Value		
				FNBG versus MNBG	FNBG versus Control	MNBG versus Control
Pain intensity (NP scale) in PACU	1.73±0.17	1.13±0.08	2.9±0.23	0.001	<0.0001	<0.0001
Morphine consumption (mg) in PACU (total)*	8.77±1.31	2.06±0.86	16.97±1.26	<0.0001	<0.0001	<0.0001
Pain at rest (VAS) (mm), POD-0	49.00±4.86	26.87±5.16	48.34±4.54	0.003	NS	0.005
Morphine consumption (PCA) (mg), POD-0	14.77±1.89	2.32±0.79	21.97±2.21	<0.0001	0.02	<0.0001
Pain at rest (VAS) (mm), POD-1	35.93±5.26	26.13±4.31	34.45±4.84	NS	NS	NS
Morphine consumption (PCA) (mg), POD-1	9.30±1.60	8.52±1.83	18.38±2.35	NS	0.003	<0.0001
Flexion (centigrade), POD-1	63.83±3.97	60.63±5.49	59.14±2.93	NS	NS	NS
Pain at flexion (VAS), POD-1	63.47±4.22	62.61±6.13	75.90±3.32	NS	0.03	0.02
Extension (centigrade), POD-1	171.67±1.56	130.23±14.17	161.90±8.27	NS	NS	NS
Pain at extension (VAS), POD-1	44.90±4.69	40.13±6.96	41.03±6.09	NS	NS	NS

All values are presented as mean±SE.

* Morphine consumption (mg) in PACU (total): morphine that was injected by method of titration in the PACU immediately after surgery followed by PCA.

FNBG, femoral nerve block group; MNBG, multiple nerve block group; NP scale, Modified Nurses’ Assessment of Postoperative Pain Scale; PACU, postanesthesia care unit; POD, postoperative day; VAS, visual analogue scale.

Prophylaxis of PONV using IV ondansetron (8 mg) before anesthesia induction was very effective. No PONV occurred in the PACU in 89 evaluated patients. One patient in the FNBG suffered nausea with no vomiting.

The duration of stay in the PACU in the MNBG (58.71±21.75 min) was significantly shorter than in the FNBG (83.33±30.69 min) and in the control group (96.14±27.24 min). A shorter duration of stay was required for patients in the FNBG than those in the control group (*P*=0.050, i.e. this difference was not statistically significant).

The intensity of pain at rest, as measured using the VAS 24 hours after surgery and 20–24 hours after discharge from the PACU (POD-0), was significantly lower in the MNBG than in the other two groups (Table 2). No difference was observed between the FNBG and the control group (Table 2). The morphine consumption by the groups was as follows: MNBG<FNBG<control group. This comparison was statistically significant (Table 2). These results were primary end-points of the study.

Fifteen patients in the FNBG, 12 patients in the MNBG, and 13 patients in the control group were treated with oral dipyrone (metamizole) or paracetamol (acetaminophen) 1–2 g orally, or non-steroidal anti-inflammatory drug (diclofenac 75 mg intramuscular) in addition to PCA IV MO due to mild pain or discomfort that generally appeared 17–24 hours after surgery.

No differences were observed between the groups regarding pain intensity at rest, range of motion (flexion and extension), or pain intensity during extension of the involved knee joint after arthroplasty at the end of POD-1 (i.e. approximately 48 hours after surgery) (Table 2). Pain intensity during flexion and morphine consumption were significantly higher in the control group than in the FNBG and MNBG on POD-1 (Table 2). The ability to walk using a walker was similar in the three groups.

No statistically significant differences were observed between the groups for any parameters on POD-2, including “Satisfaction with pain relief during the postoperative period.”

## DISCUSSION

The PROSPECT Working Group is a collaboration of anesthetists and surgeons who published a consensus with guidelines in 2008 that recommended using a single injection femoral nerve block for supplemental analgesia after TKA. These evidence-based recommendations were based on a systematic review of outcomes for TKA and other surgeries with similar pain profiles.^1^ Key and Grayling published important data on the impact of these guidelines two years later and reported that the PROSPECT recommendations “have not been universally recognized or implemented” in many institutions in South West England.^3^ Thirty-two different techniques for analgesia after TKA were used in this region of England after publication of the PROSPECT recommendations.^3^ Data from nine hospitals in Israel and other countries revealed that femoral nerve block alone was not a method of choice for supplemental analgesia after TKA. Many combinations of peripheral nerve blocks with or without infiltrative anesthesia were compared to each other, but different results were observed in the literature.^7^–^20^

We changed our practice in 2010 as a result of the PROSPECT recommendations. Ultrasound-guided single injection femoral nerve block alone was used as part of multimodal analgesia (PCA IV MO, non-steroidal anti-inflammatory drugs, and oral dipyrone or paracetamol) after TKA. We found that a preoperative single injection femoral nerve block produced only partial pain relief after TKA. Previous clinical experience with peripheral nerve blocks of the upper extremities (e.g. axillary nerve block of the four nerves in addition to intercostobrachial and medial antebrachial cutaneous nerves for regional anesthesia and postoperative analgesia after surgeries in the elbow region) demonstrated that a nerve block of all involved nerves was necessary to produce complete anesthesia and analgesia of the target region.^41^ It was therefore logical to assume that the same approach in the lower limbs would also be effective. All four nerves innervating the knee joint region should be blocked to achieve excellent analgesia during and after TKA (e.g. a combination of femoral, sciatic, obturator, and lateral femoral cutaneous nerve blocks). The benefit gained by adding the sciatic, obturator, and lateral femoral cutaneous nerve blocks to a femoral nerve block to improve postoperative analgesia and reduce opioid consumption after TKA remains controversial.^4^–^20^ Hence, comparison of the analgesic effect of femoral nerve block alone, as recommended by PROSPECT, with the effect of preoperative blocks of the femoral, sciatic, obturator, and lateral femoral cutaneous nerves was a primary end-point of our study. The compared groups were identical regarding the performance of anesthesiologists and surgeons and the surgical and anesthetic techniques used.

Significantly superior pain relief at rest and during motion, reduced morphine consumption, and a shorter stay in the PACU were demonstrated by the MNBG as compared to the FNBG and control group. Preoperative femoral nerve block alone produced only partial analgesia immediately after TKA in the PACU. Morphine consumption in the FNBG was significantly less than the control group, but all patients in the FNBG were treated with morphine. The duration of stay in the PACU was shorter in the FNBG than in the control group, but there was no statistical difference. A total of 12–15 patients (all groups) received oral dipyrone, paracetamol or non-steroidal anti-inflammatory drugs in addition to PCA IV MO.

No differences were found between the three groups on POD-2, since the duration of the blocks was over.

No complications occurred for the US-guided blocks in our small series.

Our study has several limitations. Firstly, total intravenous anesthesia using remifentanil and propofol infusions enables early awakening of the patient at the end of surgery, hence it is necessary to administer morphine 30 min before the end of surgery when remifentanil should be stopped to prevent possible acute opioid tolerance.^32^–^35^ Morphine injections at the end of surgery may result in decreased morphine consumption in the PACU. However, all patients in the three groups received the same protocol of total intravenous anesthesia and morphine (0.1 mg/kg IV) 30 min before the end of surgery. This allowed for pain intensity comparison in the PACU between groups using appropriate statistical evaluations.

Secondly, we used two scales for pain intensity measurements, the modified NP scale^38^ in the PACU and the VAS.^36^ Readers should note this difference in the presented results.

Thirdly, dipyrone, paracetamol, or non-steroidal anti-inflammatory drugs were used as needed for discomfort and/or mild pain during the postoperative period, which may have influenced pain intensity and morphine consumption.

Our main conclusions for the primary end-points of the investigation are as follows:

1. Preoperative single injection femoral nerve block alone produced partial and insufficient analgesia during and immediately after TKA, on the day of the operation, and during the first postoperative day.
2. Preoperative multiple nerve blocks of the femoral, sciatic, obturator, and lateral femoral cutaneous nerves produced excellent intra- and post-operative analgesia on the day of TKA and the first postoperative day. Patients in the MNBG had little pain immediately after surgery.
3. A significant decrease in morphine consumption during the postoperative period was noted in patients undergoing TKA in the MNBG.

Our conclusions for the secondary end-points are as follows:

1. Preinduction administration of 8 mg ondansetron (IV) was an effective prophylaxis for PONV following total intravenous anesthesia with propofol and remifentanil.
2. Time of stay in the PACU in the MNBG was significantly shorter than in other two groups.
3. Active range of motion after TKA at the end of POD-1 and during POD-2 was not affected by single injection preoperative peripheral nerve blocks, nor was the ability to walk using walker, but intensity of pain during flexion at POD-1 was higher in the control group than in the “block” groups.
4. Ultrasound guidance makes blocking of the peripheral nerves of the lower extremities before TKA safe and effective, but time is needed to perform the procedure, and the patient should be observed after the procedure. The use of a “blocking room” saves costly time in the operating room.
5. Unilateral peripheral nerve blocks enable early activity in patients on the first day after TKA.
6. Patient satisfaction with analgesia during the postoperative period as assessed after two postoperative days was similar in all groups including the control group; this result was surprising.

The main conclusion from this study is that preoperative multiple nerve blocks of the femoral, sciatic, obturator, and lateral femoral cutaneous nerves may present a more effective form of pain control and relief following TKA.

One significant limitation of preoperative single injection multiple nerve blocks for analgesia after TKA is their relatively short effect, even after the use of a long-acting local analgesic, such as bupivacaine with adrenaline. A safe adjuvant to local anesthetics for perineural or systemic use should be evaluated in the future to prolong the analgesic effect with minimal prolongation of the motor block.

Further investigation should be pursued. If confirmed, the recommendations of the PROSPECT Group should be updated.

## Abbreviations

BMI: body mass index
BW: body weight
FNB: femoral nerve block
FNBG: femoral nerve block group
MNBG: multiple nerve block group
PACU: postanesthesia care unit
PCA: patient-controlled analgesia
PCA IV MO: patient-controlled analgesia with morphine by intravenous approach
POD-0: day of operation after discharge from PACU
POD-1: first postoperative day
POD-2: second postoperative day
TKA: total knee arthroplasty
US: ultrasound
VAS: visual analogue scale

## References

[1] Fischer HBJ, Simanski CJP, Sharp C (2008). A procedure-specific systematic review and consensus recommendations for postoperative analgesia following total knee arthroplasty. Anaesthesia.

[2] Frassanito L, Vergari A, Zanghi F, Messina A, Bitondo M, Antonelli M (2010). Post-operative analgesia following total knee arthroplasty: comparison of low-dose intrathecal morphine and single-shot ultrasound-guided femoral nerve block: a randomized, single blinded, controlled study. Eur Rev Med Pharmacol Sci.

[3] Key W, Grayling M (2010). PROSPECT, before and after: anaesthesia for total knee arthroplasty. Eur J Anaesthesiol.

[4] Paul JE, Arya A, Hurlburt L (2010). Femoral nerve block improves analgesia outcomes after total knee arthroplasty: a meta-analysis of randomized controlled trials. Anesthesiology.

[5] Chan EY, Fransen M, Parker DA, Assam PN, Chua N (2014). Femoral nerve blocks for acute postoperative pain after knee replacement surgery. Cochrane Database Syst Rev.

[6] Sahin L, Korkmaz HF, Sahin M, Atalan G (2014). Ultra-sound-guided single-injection femoral nerve block provides effective analgesia after total knee arthroplasty up to 48 hours. Agri.

[7] Safa B, Gollish J, Haslam L, McCartney CJ (2014). Comparing the effects of single shot sciatic nerve block versus posterior capsule local anesthetic infiltration on analgesia and functional outcome after total knee arthroplasty: a prospective, randomized, double-blinded, controlled trial. J Arthroplasty.

[8] Abdallah FW, Chan VW, Gandhi R, Koshkin A, Abbas S, Brull R (2014). The analgesic effects of proximal, distal, or no sciatic nerve block on posterior knee pain after total knee arthroplasty: a double-blind placebo-controlled randomized trial. Anesthesiology.

[9] Yun XD, Yin XL, Jiang J (2015). Local infiltration analgesia versus femoral nerve block in total knee arthroplasty: a meta-analysis. Orthop Traumatol Surg Res.

[10] Mei S, Jin S, Chen Z, Ding X, Zhao X, Li Q (2015). Analgesia for total knee arthroplasty: a meta-analysis comparing local infiltration and femoral nerve block. Clinics (Sao Paulo).

[11] Nagafuchi M, Sato T, Sakuma T (2015). Femoral nerve block-sciatic nerve block vs. femoral nerve block-local infiltration analgesia for total knee arthroplasty: a randomized controlled trial. BMC Anesthesiol.

[12] Soltesz S, Meiger D, Milles-Thieme S, Saxler G, Ziegeler S (2016). Intermittent versus continuous sciatic block combined with femoral block for patients undergoing knee arthroplasty. A randomized controlled trial. Int Orthop.

[13] Abdallah FW, Madjdpour C, Brull R (2016). Is sciatic nerve block advantageous when combined with femoral nerve block for postoperative analgesia following total knee arthroplasty? A meta-analysis. Can J Anaesth.

[14] Grape S, Kirkham KR, Baeriswyl M, Albrecht E (2016). The analgesic efficacy of sciatic nerve block in addition to femoral nerve block in patients undergoing total knee arthroplasty: a systematic review and meta-analysis. Anaesthesia.

[15] Albrecht E, Guyen O, Jacot-Guillarmod A, Kirkham KR (2016). The analgesic efficacy of local infiltration analgesia vs femoral nerve block after total knee arthroplasty: a systematic review and meta-analysis. Br J Anaesth.

[16] Runge C, Borglum J, Jensen JM (2016). The analgesic effect of obturator nerve block added to a femoral triangle block after total knee arthroplasty: a randomized controlled trial. Reg Anesth Pain Med.

[17] Kim JH, Cho MR, Kim SO, Kim JE, Lee DK, Roh WS (2012). A comparison of femoral/sciatic nerve block with lateral femoral cutaneous nerve block and combined spinal epidural anesthesia for total knee replacement arthroplasty. Korean J Anesthesiol.

[18] Macalou D, Trueck S, Meuret P (2004). Postoperative analgesia after total knee replacement: the effect of an obturator nerve block added to the femoral 3-in-1 nerve block. Anesth Analg.

[19] Kardash K, Hickey D, Tessler MJ, Payne S, Zukor D, Velly AM (2007). Obturator versus femoral nerve block for analgesia after total knee arthroplasty. Anesth Analg.

[20] McNamee DA, Parks L, Milligan KR (2002). Post-operative analgesia following total knee replacement: an evaluation of the addition of an obturator nerve block to combined femoral and sciatic nerve block. Acta Anaesthesiol Scand.

[21] Horner G, Dellon AL (1994). Innervation of the human knee joint and implications for surgery. Clin Orthop Relat Res.

[22] Moore DC, Moore DC (1965). Block of the Lateral Femoral Cutaneous Nerve. Regional Block.

[23] Malenka RC, Nestler EJ, Hyman SE, Sydor A, Brown RY (2009). Reinforcement and Addictive Disorders. Molecular Neuropharmacology: A Foundation for Clinical Neuroscience.

[24] Beek JV, Beek JV (2009). The Femoral Block. The Neuraxiom Playbook of 9 Essential Blocks. A Handbook of Ultrasound Guided Regional Nerve Blocks.

[25] Fujiwara Y, Sato Y, Kitayama M, Shibata Y, Komatsu T, Hirota K (2007). Obturator nerve block using ultrasound guidance. Anesth Analg.

[26] Anagnostopoulou S, Kostopanagiotou G, Paraskeuopoulos T, Saranteas T (2008). Obturator nerve block: from anatomy to ultrasound guidance. Anesth Analg.

[27] Tsui BCH, Tsui BCH (2007). Popliteal Sciatic Nerve Block (Lateral Approach). Atlas of Ultrasound and Nerve Stimulation Guided Regional Anesthesia.

[28] Beek JV, Beek JV (2009). The Popliteal Sciatic Block. The Neuraxiom Playbook of 9 Essential Blocks. A Handbook of Ultrasound Guided Regional Nerve Blocks.

[29] Hurdle MF, Weingarten TN, Crisostomo RA, Psimos C, Smith J (2007). Ultrasound-guided blockade of the lateral femoral cutaneous nerve: technical description and review of 10 cases. Arch Phys Med Rehabil.

[30] Tumber PS, Bhatia A, Chan VW (2008). Ultrasound-guided lateral femoral cutaneous nerve block for meralgia paresthetica. Anesth Analg.

[31] Tramèr MR, Reynolds DJ, Moore RA, McQuay HJ (1997). Efficacy, dose-response, and safety of ondansetron in prevention of postoperative nausea and vomiting: a quantitative systematic review of randomized placebo-controlled trials. Anaesthesiology.

[32] Casati A, Cappelleri G, Berti M, Fanelli G, Di Benedetto P, Torri G (2002). Randomized comparison of remifentanil-propofol with a sciatic-femoral nerve block for out-patient knee arthroscopy. Eur J Anaesthesiol.

[33] Schmidt J, Hering W, Albrecht S (2005). Anaesthesist.

[34] Joly V, Richebe P, Guignard B (2005). Remifentanil-induced postoperative hyperalgesia and its prevention with small-dose ketamine. Anaesthesiology.

[35] Hansen EG, Duedahl TH, Rømsing J, Hilsted KL, Dahl JB (2005). Intra-operative remifentanil might influence pain levels in the immediate post-operative period after major abdominal surgery. Acta Anaesthesiol Scand.

[36] Wewers ME, Lowe NK (1990). A critical review of visual analogue scales in the measurement of clinical phenomena. Res Nurs Health.

[37] Suehiro K, Okutai R (2011). Duration of cerebral desaturation time during single-lung ventilation correlates with mini mental state examination score. J Anesth.

[38] Chung IS, Sim WS, Kim GS (2001). Nurses’ assessment of postoperative pain: can it be alternative to patients self-report?. J Korean Med Sci.

[39] Aubrun F, Mazoit J-X, Riou B (2012). Postoperative intra-venous morphine titration. Br J Anaesth.

[40] Aldrete JA (1995). The post-anesthesia recovery score revisited. J Clin Anesth.

[41] Stav A, Reytman L, Stav M-Y (2016). Comparison of the supraclavicular, infraclavicular and axillary approaches for ultrasound-guided brachial plexus block for surgical anesthesia. Rambam Maimonides Med J.

